# Simultaneous Renal Cell Carcinoma and Giant Retroperitoneal Liposarcoma Involving Small Intestine

**DOI:** 10.1155/2016/6021909

**Published:** 2016-08-09

**Authors:** Aleksandr A. Reznichenko

**Affiliations:** Division of Transplant Surgery, Department of Surgery, University of Cincinnati, 231 Albert Sabin Way, Suite 1555, Cincinnati, OH 45267-0519, USA

## Abstract

*Background*. The concomitant occurrence of a renal cell carcinoma and retroperitoneal sarcoma is extremely rare with only few cases being reported.* Methods*. We present a case of simultaneous renal cell carcinoma and exceptionally large size retroperitoneal sarcoma involving small intestine. Surgical resection of retroperitoneal sarcoma and simultaneous right nephrectomy were performed.* Results*. Patient developed recurrent and metastatic disease and underwent debulking surgery following by chemotherapy. Despite aggressive behavior of the retroperitoneal sarcomas, patient is currently (7 years after simultaneous resection and nephrectomy) recurrence-free.* Conclusions*. Complete surgical resection is the mainstay of therapy for both renal cell carcinoma and retroperitoneal sarcoma. We present a case of simultaneous renal cell carcinoma and exceptionally large size retroperitoneal sarcoma. Debulking surgery and chemotherapy were helpful in our case.

## 1. Case Presentation

**Figure 1:**
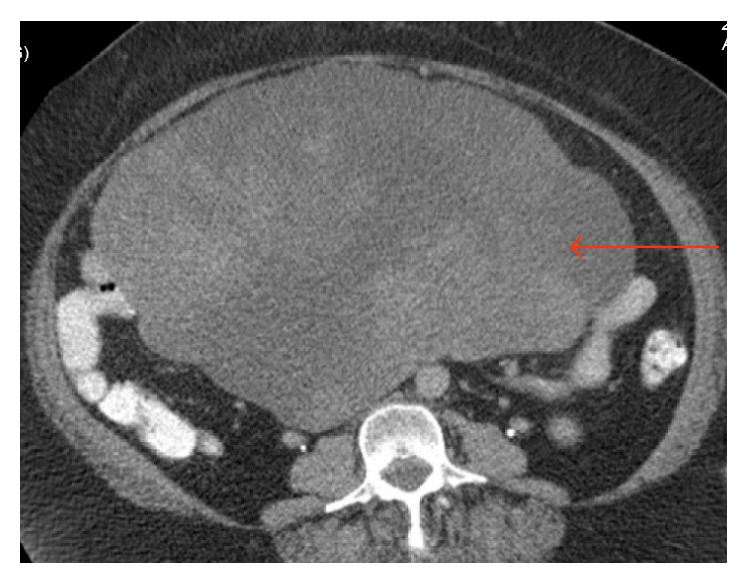
Abdomen CT scan with IV and PO contrast, axial view. Large multilobular mass (red arrow).

**Figure 2:**
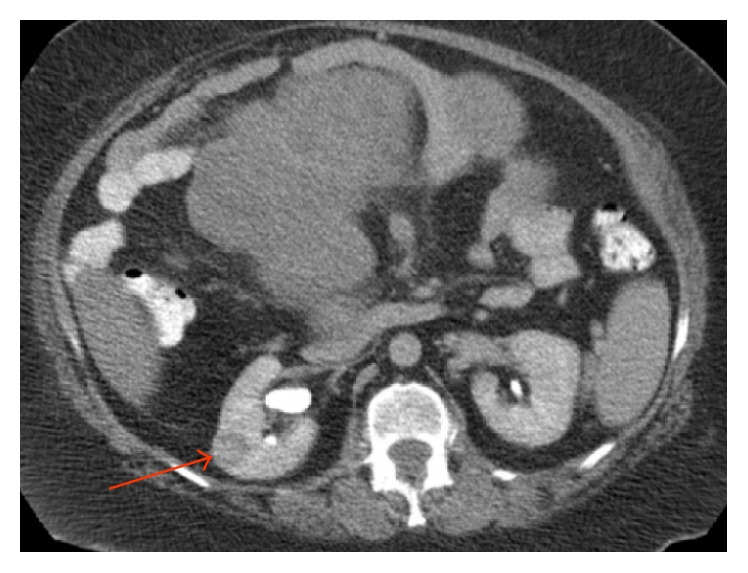
Abdomen CT scan with IV and PO contrast, axial view. Solid mas in the right kidney (red arrow).

**Figure 3:**
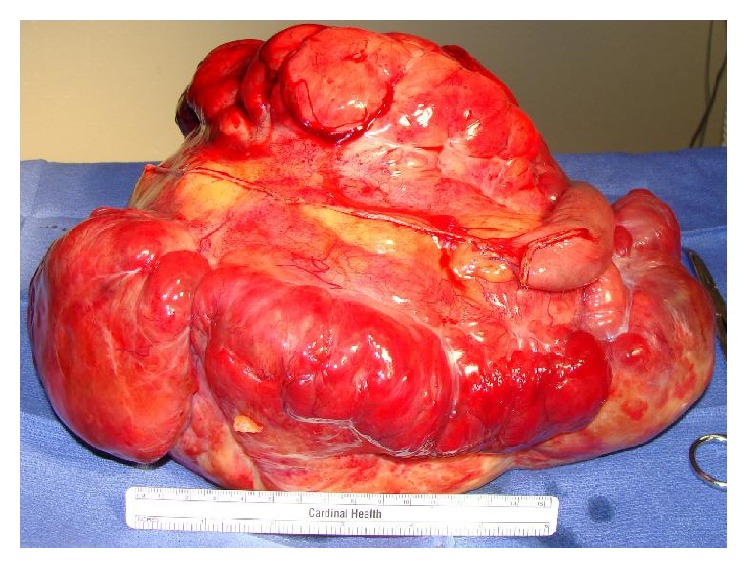
Surgical specimen: large retroperitoneal sarcoma involving small bowel and mesentery.

A 61-year-old woman with history of morbid obesity, open cholecystectomy, and sarcoma excision from right lower extremity and from left buttock (6 and 3 years ago, resp.) developed fatigue, abdominal pain, and large palpable mass occupying entire abdomen and right flank. A computer tomography (CT) scan showed 27 × 20 cm multilobular abdominal mass and 2.5 × 2.5 cm solid mass in the middle pole of the right kidney (Figures 1 and 2). Laboratory data was unremarkable, and serum creatinine was 0.7 mg/dL. Patient underwent exploratory laparotomy, resection of large retroperitoneal tumor (weight 5621 gm) en block with small intestine (Figure 3), and primary small bowel to small bowel anastomosis. The mass in right kidney was resected and sent for frozen section, which revealed a malignant neoplasm of uncertain etiology. Right nephrectomy was performed. Final pathology showed renal cell carcinoma and myxoid liposarcoma involving small bowel and mesentery, with clear resection margins. Patient had uneventful recovery.

Eight months later, the patient developed intraperitoneal recurrence of sarcoma and underwent debulking surgery.

Subsequently, she developed recurrent and metastatic disease involving spleen, mesentery, liver, pelvis, and lungs and received multiple courses of chemotherapy, including Gemcitabine, Taxotere, and a clinical trial of Yondelis. Following the chemotherapy treatment described above, the patient had near complete resolution of all intra-abdominal and pulmonary nodules and currently (7 years after resection of retroperitoneal mass and nephrectomy) is recurrence-free.

Liposarcoma is one of the most common soft tissue sarcomas found in adults. It has a predilection for retroperitoneal space. Renal cell carcinoma is the most common tumor of the kidney [1]. Patients with primary malignant fibrous histiocytoma demonstrate a risk for developing a renal cell carcinoma [2].

The concomitant occurrence of a renal cell carcinoma and retroperitoneal sarcoma is extremely rare with only few cases being reported [3, 4].

Surgical resection is the mainstay of therapy for both renal cell carcinoma and retroperitoneal sarcoma [1, 3, 4].

This case is noticeable because of the good outcome in our patient despite extremely aggressive behavior of the tumor and also because of exceptionally large size of retroperitoneal sarcoma.
